# Prediction of Tacrolimus–Posaconazole Interactions in Renal Transplant Patients with Different CYP3A5 Genotypes, Based on Physiological Pharmacokinetic Models

**DOI:** 10.3390/pharmaceutics18060639

**Published:** 2026-05-22

**Authors:** Mengmeng Guan, Wanyi Zhou, Haoran Qin, Yi Xu, Di Zhao, Hui Xue, Nan Hu

**Affiliations:** 1 Department of Pharmacy, The Third Affiliated Hospital of Soochow University, Changzhou 213000, China; 20235226106@stu.suda.edu.cn (M.G.); 20245226013@stu.suda.edu.cn (W.Z.); 20235226137@stu.suda.edu.cn (H.Q.); 20245226129@stu.suda.edu.cn (Y.X.); 2 College of Pharmaceutical Sciences, Soochow University, Suzhou 215000, China; 3 School of Basic Medicine and Clinical Pharmacy, China Pharmaceutical University, Nanjing 210000, China; zh_d99@cpu.edu.cn; 4 Department of Pharmacy, The First People’s Hospital of Changzhou, Changzhou 213000, China

**Keywords:** physiologically based pharmacokinetic, CYP3A5, tacrolimus, posaconazole, drug–drug interactions

## Abstract

**Objective:** Posaconazole, a second-generation triazole antifungal used for the prevention or treatment of invasive fungal infections, has been shown to markedly increase tacrolimus exposure in vivo when co-administered, potentially leading to clinically significant adverse events. A physiologically based pharmacokinetic (PBPK) model was developed to predict tacrolimus–posaconazole interactions in renal transplant recipients with different CYP3A5 genotypes, to inform tacrolimus dose adjustment in clinical practice. **Methods:** First, to obtain the critical inhibition parameters, in vitro enzyme kinetic studies were conducted. Based on these data, a whole-body physiologically based pharmacokinetic (PBPK) model for TAC was developed and validated in PK-Sim. A published, validated posaconazole PBPK model was applied concurrently. Model performance was evaluated against published pharmacokinetic data in healthy volunteers receiving tacrolimus with posaconazole. A virtual Chinese renal transplant recipient was generated by incorporating population-specific physiological parameters, including CYP3A5 genotype-dependent enzyme expression. **Results:** In vitro experimental results demonstrated that POSA acts as a potent reversible competitive inhibitor of CYP3A4/5-mediated TAC metabolism. The tacrolimus PBPK model adequately captured pharmacokinetics across CYP3A5 genotypes, and tacrolimus pharmacokinetics during co-administration with posaconazole were also predicted. Compared with CYP3A5 expressers, nonexpressers showed greater variability in tacrolimus whole-blood concentrations and greater susceptibility to posaconazole-mediated interactions. The CYP3A5*3*3 genotype was associated with higher C_max_ and AUC. Dose optimization simulations predicted that after 6–7 days of posaconazole co-administration, nonexpressers would require the reduction of tacrolimus dosing frequency from every 12 h to every 24 h to maintain trough concentrations within 8–15 ng/mL, whereas a 50% dose reduction was predicted to be optimal for expressers. **Conclusions:** A tacrolimus–posaconazole PBPK drug–drug interaction model was developed for the population of renal transplant recipients and used to simulate tacrolimus trough concentrations across CYP3A5 genotypes and dosing regimens, supporting genotype-informed co-administration in clinical practice.

## 1. Introduction

Renal transplantation is considered the most effective treatment for end-stage renal disease and has been associated with improved survival and quality of life. Tacrolimus (TAC) is widely used as first-line immunosuppressive therapy after renal transplantation [1] However, TAC has a narrow therapeutic window, and its pharmacokinetics are affected by multiple factors. Substantial inter- and intra-individual variability in TAC exposure is therefore observed, which may compromise efficacy and increase the risk of adverse events. Subtherapeutic TAC concentrations may increase the risk of rejection, whereas supratherapeutic concentrations may increase the risk of adverse drug reactions, including nephrotoxicity [2]. TAC is primarily metabolized by cytochrome P450 (CYP) enzymes in the intestine and liver, mainly CYP3A5 and CYP3A4 [3].

The influence of CYP3A5 polymorphisms on TAC metabolism in vivo has been widely reported. CYP3A5 polymorphisms have been estimated to explain 40–50% of the variability in TAC dose requirements. The most extensively studied CYP3A5 variant is CYP3A5*3 (rs776746), an A-to-G transition at position 6986 in intron 3. This variant induces aberrant splicing and produces a truncated protein, thereby reducing CYP3A5 activity [4]. Accordingly, compared with CYP3A5 nonexpressers (CYP3A5*3*3), CYP3A5 expressers (CYP3A5*1*1 or *1*3) have lower dose-normalized trough concentrations (C_min_). In the Chinese population, the CYP3A5*3 allele frequency has been reported to be as high as 77.8%. Therefore, the role of CYP3A5 in TAC dose individualization warrants further investigation [5].

Solid organ transplant recipients are at increased risk of invasive fungal disease (IFD) because of long-term immunosuppressive therapy. IFD is an important cause of morbidity and mortality in solid organ transplant recipients [6,7]. Posaconazole (POSA) is a systemic, second-generation triazole antifungal with broad-spectrum activity against multiple fungal pathogens. POSA has been shown to reduce the risk of IFD in transplant recipients and is used for both prophylaxis and treatment [8]. POSA is a CYP3A inhibitor that can inhibit TAC metabolism. Co-administration has been associated with increased TAC whole-blood concentrations [9,10,11]; therefore, TAC dose reductions (e.g., to approximately one-third) have been recommended [12]. However, existing labeling and published studies provide broad recommendations, and formal, individualized dose-adjustment guidance remains limited. The effect of POSA co-administration on TAC whole-blood concentrations and the concentration-to-dose ratio in renal transplant recipients has previously been evaluated, and patient genetic polymorphisms were identified as important determinants of interaction magnitude [13]. Although these findings improved understanding of the POSA–TAC interaction, more quantitative approaches are needed to guide TAC administration. To date, no official dose-adjustment guideline is available for POSA–TAC co-administration.

Physiologically based pharmacokinetic (PBPK) models integrate drug-specific data with human physiology, incorporating metabolic enzymes and transporters to predict drug–drug interaction (DDI) outcomes. PBPK modeling has been increasingly used to predict DDI [14]. PBPK-based DDI models involving TAC or POSA with other drugs have been reported; however, a model that explicitly captures the TAC–POSA interaction remains unavailable. Therefore, a PBPK model of the TAC–POSA DDI was developed and used to simulate TAC concentration–time profiles across CYP3A5 genotypes and dosing regimens to support clinical TAC dosing.

An important prerequisite for establishing a PBPK model of drug interactions is to obtain a reliable enzyme inhibition constant (K_i_), a value which quantitatively defines the DDI potential. As there is no research on the inhibition of POSA on the CYP3A5 enzyme, and the K_i_ value is highly substrate-dependent, this gap may introduce uncertainty when the target substrate is TAC. Therefore, before the model was constructed, we performed in vitro enzyme kinetics experiments using CYP3A4/5 recombinase and TAC as probe substrates. The main purpose of these experiments was to determine the half inhibitory concentration (IC_50_) and apparent K_i_ of POSA on CYP3A4 and CYP3A5, and then integrate the parameters determined by these experiments into the PBPK model.

## 2. Materials and Methods

### 2.1. Reagents and Chemicals

TAC (batch no. 12-MWC-10-1; purity, 98%), ascomycin (batch no. 1-OBI-122-1; purity, 95%), POSA (batch no. 3-JLW-3-1; purity, 98%), and ketoconazole (batch no. 8-MIC-131-1; purity, 98%) were purchased from Toronto Research Chemicals (Toronto, ON, Canada). Human recombinant CYP3A4/5 enzyme was obtained from Shanghai Rild Liver Research Institute Co., Ltd (Shanghai, China). β-nicotinamide adenine dinucleotide phosphate (β-NADP), glucose-6-phosphate (G-6-P), and glucose-6-phosphate dehydrogenase (G-6-PD) were purchased from Sigma-Aldrich (St. Louis, MO, USA), and anhydrous magnesium chloride was purchased from Innochem (Beijing, China). Chromatographically pure methanol and acetonitrile were acquired from Merck (Darmstadt, Germany); analytic-grade pure formic acid and amine acetate were acquired from Sigma-Aldrich; pure water was produced from the Milli-Q ultrapure water system.

### 2.2. LC-MS/MS Detection Method

TAC concentrations were quantified by LC-MS/MS, using established methods [15], which were fully validated in accordance with the ICH M10 guideline on bioanalytical method validation [16]. The validated method met the acceptance criteria for accuracy, precision, selectivity, sensitivity, and stability, as defined in the guideline. Detailed descriptions of the LC-MS/MS detection method and the sample preparation procedure, in addition to the full validation results, are provided in the Supplementary Materials.

### 2.3. Study on Recombinant Enzyme In Vitro

#### 2.3.1. Preparation of NADPH Regeneration System

First, 15.31 mg β-NADP was weighed and dissolved in 2 mL PBS to prepare solution A (10 mmol/L). Second, 6.08 mg G-6-P was accurately weighed and dissolved in 0.2 mL PBS to obtain solution B (100 mmol/L). Third, G-6-PD (1000 U) lyophilized powder was reconstituted with 1 mL ultrapure water to yield a 1000 U/mL stock; 10 μL of this stock was mixed with 990 μL PBS to prepare solution C (10 U/mL). Finally, 38.08 mg MgCl_2_ was dissolved in 10 mL PBS to prepare solution D (40 mmol/L). Immediately prior to use, the four solutions were mixed in appropriate proportions to afford a complete NADPH-regenerating system with final concentrations of β-NADP 1 mmol/L, G-6-P 10 mmol/L, MgCl_2_ 4 mmol/L, and G-6-PD 1 U/mL in the incubation mixture.

#### 2.3.2. Determination of the IC_50_ Value of Posaconazole for CYP3A4/5 Enzymes

After preparing the PBS and the solutions of G-6-P, G-6-PD, and MgCl_2_ described in Section 2.3.1, mix them in a ratio of 8:2:2:2. Add 140 μL of the mixed solution to each Eppendorf tube. Add 20 μL of CYP3A4/5 enzyme solution (2 pM), and then add 10 μL of posaconazole inhibitor solution to achieve final concentrations of 0.001, 0.01, 0.1, 0.5, 1, 2, 5, 10 and 20 μM. Mix and preincubate at 37 °C for 5 min. Preincubate the prepared β-NADP solution under the same conditions for 5 min. Add 10 μL of tacrolimus substrate solution (1.0 µM), and then initiate the reaction by adding 20 μL of 10 mmol/L β-NADP solution and immediately incubate at 37 °C. After incubation for 10 min and 5 min (respectively, for the two time points), terminate the reactions by adding 400 μL of precooled methanol. Add 5 μL internal standard solution. Vortex the quenched samples for 1 min, mix thoroughly, centrifuge, and inject the supernatant for analysis. Test each concentration in three replicate wells. Calculate residual enzyme activity as the ratio of substrate depletion in the inhibitor group to that in the blank control. Plot residual activity versus the log-transformed inhibitor concentration to estimate the IC_50_.

#### 2.3.3. The Calculation of Ki

In the present PBPK model, the inhibition of CYP3A5 by posaconazole was assumed to be competitive. This assumption was adopted because the posaconazole model used here was derived from the validated PBPK model published by Gerner et al. [17], in which posaconazole is explicitly characterized as a competitive inhibitor of CYP3A4, with an assigned Kᵢ. A comprehensive search of the literature yielded no in vitro study defining the inhibition type associated with posaconazole toward CYP3A5 specifically. However, it has been clearly demonstrated that fluconazole—a triazole antifungal structurally related to posaconazole—inhibits human recombinant CYP3A5 in a competitive manner, and is associated with a reported Kᵢ value [18]. Given the well-recognized overlap in CYP3A inhibition profiles among triazole antifungals, we considered competitive inhibition of CYP3A5, which is analogous to CYP3A4, to be the most conservative and mechanistically plausible assumption. Therefore, the Cheng–Prusoff equation (Equation (1)) is used to convert the experimentally measured values into inhibition constants.

(1) $$K_{i}=IC_{50}/(1+[S]/K_{m})\;$$

Among these terms, IC_50_ is the half inhibitory concentration determined by the experiment; [S] is the final concentration of the substrate tacrolimus in the incubation system; and K_m_ is the Michaelis constant of tacrolimus in the recombinant CYP3A4 or CYP3A5 enzyme system.

In the preliminary kinetic experiment of this study, we pre-determined the K_m_ values of tacrolimus on CYP3A4 and CYP3A5 under the optimized enzyme concentration and incubation time conditions (the optimization process and results are detailed in the Supplementary Materials). The measured K_m_ values (CYP3A4 was 1.438 μM, CYP3A5 was 1.199 μM) and the corresponding IC_50_ values were substituted into Formula 1 to calculate the K_i_ values of posaconazole for the two subtypes of enzymes.

### 2.4. PBPK Model Development and Evaluation

#### 2.4.1. Model Development and Validation

A TAC PBPK model was developed using a bottom-up approach, based on published works in the literature. For initial model development, an extensive search of the literature was conducted to obtain the physicochemical properties of TAC, including molecular weight, lipophilicity, pKa, and solubility. Clinical pharmacokinetic data for TAC and POSA model development were obtained from published studies. In PK-Sim, the default CYP3A5 concentration is 0.04 µmol/L, representing expression levels in CYP3A5 nonexpressers. To represent CYP3A5 expressers, the CYP3A5 concentration was set to 0.60 µmol/L (Caucasian) [19]. Because TAC is primarily metabolized hepatically and eliminated via bile, with <1% excreted in urine [20], renal elimination was not included. Key parameters governing TAC biotransformation, including K_m_ and K_cat_, were optimized using the parameter identification module in PK-Sim and observed clinical pharmacokinetic (PK) data. The model’s parameters are summarized in Table 1.

First, TAC-related parameters were fitted to clinical PK data after intravenous administration to optimize the kinetic parameters of CYP3A4. Subsequently, oral PK data were used to optimize absorption-related parameters, including intestinal permeability. Because CYP3A expression differs by ethnicity, with Asian populations reported to have CYP3A4 and CYP3A5 expression levels of 0.90-fold and 0.88-fold (vs. Caucasians) [24], an Asian TAC PBPK model was established by adjusting in vivo CYP3A5 expression to fit single-dose oral TAC PK data in Asian CYP3A5 expressers and nonexpressers.

The POSA PBPK model was adopted from published works in the literature [17]. As a CYP3A inhibitor, POSA was assumed to increase TAC exposure by inhibiting CYP3A-mediated metabolism. POSA is metabolized primarily via UGT1A4. The CYP3A5 inhibition constant (K_i_) for POSA, obtained from the in vitro recombinant enzyme assays, was incorporated into the POSA PBPK model. The POSA model’s parameters are summarized in Table 2.

For each included study, a representative virtual individual was established based on the means and modes for the age, sex, weight, height, body mass index, and ethnicity of the respective study populations. If demographic information was incomplete, default settings were adopted from the population database accessible in PK-Sim. A virtual population of 1000 individuals was created for each study population, using the respective reported demographic information to visually assess variability based on these characteristics relative to the metabolizing of enzymes. If no relevant data were available, an age range of 20–50 years was assumed. In the absence of genotype/phenotype information for a particular study group, CYP3A5 activity was assumed according to the frequency of the *1 allele observed in the respective ethnic group, as published by Birdwell et al.

Predictive performance was evaluated by comparing observed and predicted clinical PK data. The fold error between the predicted and measured PK parameters, including the area under the plasma concentration–time curve (AUC), maximum blood concentration (C_max_), and the average folding error (AFE) of all concentration–time data points.

(2) $$\mathrm{Error}=\frac{\mathrm{Predicted}}{\mathrm{Observed}}$$

(3) $$AFE=10^{\frac{1}{n}\sum_{i=1}^{n}\left|\log_{10}\left(\frac{C_{\mathrm{pred},i}}{C_{\mathrm{obs},i}}\right)\right|}$$

where “predicted” represents the predicted value and “observed” represents the measured or observed value. The model predictions were considered acceptable if the fold error was between 0.5 and 2.0.

#### 2.4.2. DDI Model Development and Validation

After the single-drug pharmacokinetics were adequately captured, the TAC and POSA models were further evaluated using clinical DDI data to confirm their applicability for assessing the DDI, with POSA as the precipitant and TAC as the object drug. For validation, simulated dosing regimens and sampling schedules were matched to the corresponding clinical DDI studies in healthy volunteers. The TAC model was evaluated as an object drug using clinical DDI data for TAC co-administered with voriconazole, a CYP3A inhibitor. The POSA model was evaluated as a precipitant using clinical DDI data for POSA co-administered with midazolam, a sensitive CYP3A substrate. DDI magnitude was quantified using predicted-to-observed ratios for AUC and C_max_ (alone vs. co-administration) and compared with observed DDI PK data. Successful prediction was defined as predictions within a 0.5- to 2-fold range of the observed data. At the same time, quantitative model performance metric–average fold error (AFE) was used to evaluate the prediction performance of the model, and AFE ≤ 2.0 was the key criterion for the acceptable prediction of the model.

The validated TAC and POSA PBPK models were integrated to construct the TAC–POSA DDI model. A single-center, open-label PK study in healthy volunteers investigated the effect of POSA on TAC pharmacokinetics [11]. PK data from this study were used to validate the predictive performance of the TAC–POSA DDI PBPK model.

#### 2.4.3. DDI Modeling and Dosing Guidance

To simulate TAC–POSA DDI in renal transplant recipients, a virtual individual representing post-transplant physiology was constructed. This approach has been used previously in renal transplant recipients; for example, Emoto et al. [27] and Maruyama et al. [28] incorporated physiological parameters (e.g., hematocrit, serum creatinine, and serum albumin) into Simcyp. However, in PK-Sim, these parameters cannot be directly modified in healthy individuals. In PK-Sim, specifying chronic kidney disease (CKD) and a target eGFR updates glomerular filtration rate (GFR), kidney volume, renal blood flow, plasma protein binding, hematocrit, gastric emptying time, and intestinal transit time. Therefore, the virtual individual was defined as CKD with a target eGFR of 31.22 mL/min/1.73 m^2^. Parameter values were derived from baseline physiological data used in our previously developed TAC population pharmacokinetic model describing renal transplant recipients receiving POSA. Subsequently, CYP3A5 concentrations were adjusted to represent CYP3A5 expressers or nonexpressers, and whole-blood TAC concentrations were simulated under different dosing regimens with or without POSA.

### 2.5. Software and Data Processing and Graphics Software

The PBPK model was developed in PK-Sim (version 12.1), part of the Open Systems Pharmacology Suite (OSP; Bayer Technology Services, Leverkusen, Germany). Clinical PK data were digitized from published figures using WebPlotDigitizer (version 4.3; Ankit Rohatgi, Pacifica, CA, USA). The IC_50_ was analyzed using GraphPad Prism (version 10.2.1). Plots were generated in RStudio (version 2023.12.1+402).

## 3. Results

### 3.1. In Vitro Recombinant Enzyme Study

**Figure 1:**
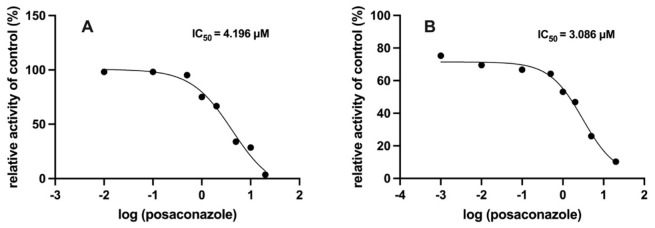
Concentration-dependent inhibition of tacrolimus metabolism by posaconazole in CYP3A4/5 recombinase. (**A**) Inhibition of CYP3A4-mediated tacrolimus metabolism. (**B**) Inhibition of CYP3A5-mediated tacrolimus metabolism. The relative metabolic activity (expressed as a percentage of the control activity in the absence of inhibitor) is plotted against the logarithm of posaconazole concentration. The solid curves represent the best-fit lines obtained by nonlinear regression using GraphPad Prism software (10.2.1). The calculated half-maximal inhibitory concentration (IC_50_) values are indicated in each panel.

To quantitatively assess the inhibitory potency of posaconazole against CYP3A4 and CYP3A5 isoforms, in vitro inhibition assays were conducted using tacrolimus as the probe substrate. The substrate concentration was fixed at 1.0 μM for the IC_50_ determination. Posaconazole exhibited concentration-dependent inhibition of tacrolimus metabolism in both isoforms. In vitro inhibition experiments showed that posaconazole had a concentration-dependent inhibitory effect on the metabolism of tacrolimus. As shown in Figure 1, by fitting the dose–response curve, we measured the IC_50_ of posaconazole on CYP3A4- and CYP3A5-mediated tacrolimus; the metabolism values were 4.196 ± 0.14 μM and 3.086 ± 0.09 μM, respectively.

Assuming a competitive inhibition mechanism, which is characteristic of azole antifungals, the inhibition constants (K_i_) were calculated using the Cheng–Prusoff equation. (Equation (2)). The Michaelis–Menten constants (K_m_) for tacrolimus, determined in our preliminary kinetic studies, were 1.438 μM for CYP3A4 and 1.199 μM for CYP3A5. Based on these parameters, the calculated K_i_ values were 2.47 μM for CYP3A4 and 1.68 μM for CYP3A5. These results indicate that CYP3A5 is slightly more sensitive to posaconazole inhibition, compared to CYP3A4, under the tested conditions.

### 3.2. Development and Evaluation of TAC and POSA Models

**Figure 2:**
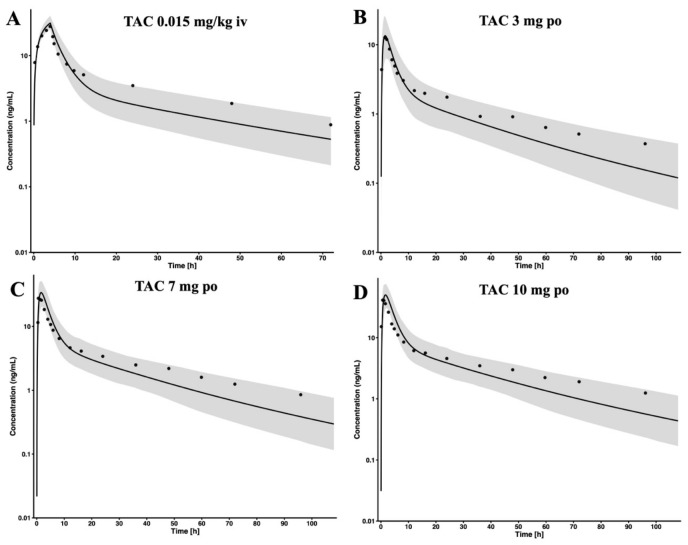
Predicted and observed plasma concentration–time profiles for tacrolimus. The solid lines represent the predicted mean tacrolimus concentration curves; the solid points represent the observed values of tacrolimus; the shaded areas represent the 5th-to-95th percentile predicted ranges; (**A**) tacrolimus 0.015 mg/kg intravenously; (**B**–**D**) tacrolimus 3, 7, 10 mg orally.

**Figure 3:**
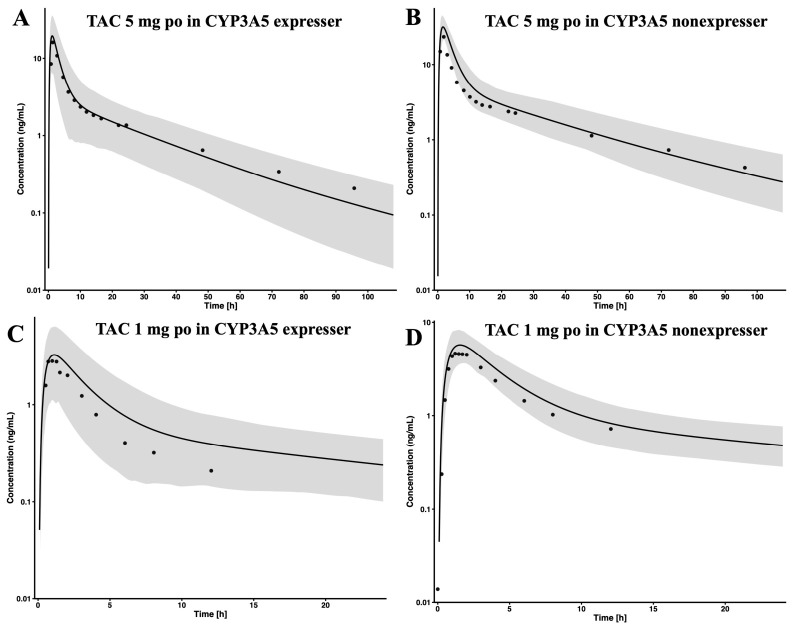
Predicted and observed plasma concentration–time profiles for tacrolimus. The solid lines represent the predicted mean tacrolimus concentration curves; the solid points represent the observed values of tacrolimus; the shaded areas represent the 5th-to-95th percentile predicted ranges; (**A**,**C**) oral tacrolimus in the CYP3A5 expressers; (**B**,**D**) oral tacrolimus in the CYP3A5 nonexpressers.

A PBPK model for TAC was developed and validated using clinical whole-blood concentration–time data. Details of the dataset, study population characteristics, and formulation are summarized in Table 3. A PK dataset from healthy volunteers receiving single intravenous and oral doses was used for model development. To assess the impact of CYP3A5 genotype on TAC exposure, TAC PK data were obtained in CYP3A5 expressers and nonexpressers. Asian CYP3A5 expressers (CYP3A5*1*3 or *1*1) and nonexpressers (CYP3A5*3*3) were assigned hepatic CYP3A5 concentrations of 0.53 and 0.04 μmol/L, respectively. As shown in Figure 2 and Figure 3, the simulated mean concentration–time profile of tacrolimus showed good agreement with the observed PK data, and the observed values were almost within the 5th-to-95th percentile probability line of the simulated curve. At the same time, in order to verify the predictive performance of the model, we summarized the pharmacokinetic parameters of tacrolimus in TAC clinical studies, as shown in Table 4. The AUC_0-∞_ ratio of tacrolimus was 0.71 to 1.87, and the predicted/observed ratio of C_max_ ratio was between 0.5 and 1.5. All the predicted values fell within the 2-fold error range of the observed values, indicating that the model can reliably predict the pharmacokinetics of TAC.

The POSA PBPK model reported by Gerner et al. was used. Clinically used POSA formulations include an oral suspension and delayed-release tablets; POSA is lipophilic, and its absorption is substantially affected by food. Therefore, PK data under different formulations and feeding conditions were used for model evaluation. Simulated POSA concentrations consistently fell within a 0.5- to 2-fold range of the observed values. At the same time, we calculated the average folding error (AFE) of the predicted and observed concentrations of POSA and TAC. The calculation results show that AFE ≤ 2.0, indicating the accuracy of the model prediction.

### 3.3. Development and Validation of the DDI Model

We previously validated the TAC and POSA PBPK models using clinical PK data, with simulated concentrations within a 0.5- to 2-fold range of the observed values. Given that the objective was to develop a CYP3A inhibition-mediated DDI model, we simulated two benchmark DDIs: TAC (substrate) with voriconazole (inhibitor), and POSA (inhibitor) with midazolam (substrate). Model performance for the TAC (substrate) and POSA (inhibitor) was evaluated by comparison with published clinical DDI PK data. For both DDIs, predicted AUC and C_max_ values fell within a 0.5- to 2-fold range of the observed values (Table 5).

Gerner et al. previously simulated the effect of POSA on intravenous midazolam PK and estimated the key parameter, the CYP3A4 inhibition constant (K_i_). Using a value from the literature of 0.42 μM as the initial estimate, the optimized K_i_ was 5.22 × 10^−3^ μM. This optimized value was adopted in the POSA model used in the present study. In addition, based on the in vitro IC_50_ values obtained in this study, the CYP3A5 inhibition constant was calculated as 3.84 × 10^−3^ μM and implemented in the TAC–POSA DDI model.

In order to evaluate the accuracy of this model in predicting posaconazole–tacrolimus interaction, we used key clinical study data reported in the literature for verification. This study investigated the effect of posaconazole on the pharmacokinetics of tacrolimus in healthy volunteers. As shown in Table 5, the model successfully predicted a significant increase in tacrolimus exposure after combination with posaconazole. The model predictions of tacrolimus AUC_0–t_ Ratio and C_max_ Ratio were highly consistent with the clinical observations, and the predicted/observed ratios were 1.14 and 1.00, respectively. All the predicted values fall within two-times the error range of the observed values. Quantitative evaluation showed that the average folding error (AFE) predicted by the main PK parameters was less than 2, indicating that the model prediction had no significant deviation and good accuracy. The fitting of the drug–time curve, as predicted by the model with the observed data points, is shown in the Supplementary Materials.

### 3.4. DDI Model Simulation and Dosing Guidance

**Figure 4:**
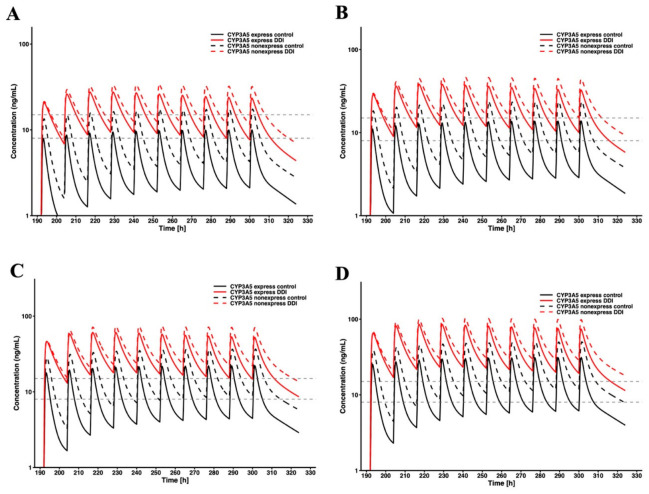
Simulated steady-state blood concentration–time profiles of tacrolimus with or without posaconazole co-administration in different CYP3A5 genotypes ((**A**) tacrolimus 0.075 mg/kg/d; (**B**) tacrolimus 0.1 mg/kg/d; (**C**) tacrolimus 0.15 mg/kg/d; and (**D**) tacrolimus 0.2 mg/kg/d). The grey dotted line represents the target therapeutic range of tacrolimus (8–15 ng/mL) in the early stage after transplantation.

Virtual individuals were constructed for CYP3A5 expressers and nonexpressers, with CKD specified and a target eGFR of 31.22 mL/min/1.73 m^2^. According to an expert consensus on individualized TAC therapy after solid organ transplantation, the recommended TAC dose for renal transplant recipients is 0.075–0.25 mg/kg/day, administered every 12 h. We therefore simulated steady-state TAC concentrations with and without POSA co-administration across CYP3A5 genotypes at TAC doses of 0.075, 0.10, 0.15, and 0.20 mg/kg/day. The results are shown in Figure 4 and Table 6. Compared with CYP3A5 expressers, nonexpressers showed greater variability in TAC concentrations and were more sensitive to POSA co-administration. CYP3A5*3*3 carriers showed higher C_max_ and AUC. Accordingly, greater TAC dose reduction was required in CYP3A5 nonexpressers, during co-administration with POSA, to achieve target concentrations.

**Figure 5:**
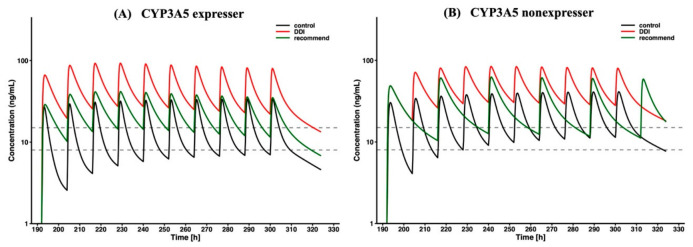
Simulated blood concentration–time profiles for tacrolimus after tacrolimus multiple administration in different CYP3A5 genotypes ((**A**) CYP3A5 expressers; (**B**) CYP3A5 nonexpressers). (**A**) Black solid line: TAC 0.2 mg/kg/d; red solid line: TAC 0.2 mg/kg/d + POSA 200 mg tid; and green solid line: TAC 0.1 mg/kg/d + POSA 200 mg tid. (**B**) Black sold line: TAC 0.15 mg/kg/d; red solid line: TAC 0.15 mg/kg/d + POSA 200 mg tid; and green solid line: TAC 0.15 mg/kg/48 h + POSA 200 mg tid. The grey dotted line represents the target therapeutic range of tacrolimus (8–15 ng/mL) in the early stage after transplantation.

We also performed TAC dose-optimization simulations during co-administration with POSA. The results are shown in Figure 5. Simulations indicated that POSA reached a steady state after approximately 6–7 days of co-administration. In CYP3A5 nonexpressers, while keeping the TAC dose per administration unchanged, extending the dosing interval from every 12 h to every 24 h was required to maintain target whole-blood trough concentrations (8–15 ng/mL). In CYP3A5 expressers, reducing the TAC dose to 50% of the baseline dose was sufficient during co-administration.

## 4. Discussion

In clinical practice, combination therapy is increasingly common, and drug–drug interactions are a critical determinant of safety and efficacy. Traditional DDI studies based on clinical trials are costly and time-consuming and may raise ethical concerns. Accordingly, physiologically based pharmacokinetic modeling has been widely adopted for quantitative DDI prediction. PBPK models integrate drug physicochemical properties, human physiology/anatomy, and in vitro enzyme/transporter data to construct virtual individuals. This framework enables mechanistic simulation of absorption, distribution, metabolism, and excretion, supporting prospective assessment of DDI risk [33]. Regulatory agencies may accept PBPK simulations, when adequately verified and validated for the intended purpose, to inform labeling and reduce the need for dedicated clinical DDI studies [34]. Common PBPK platforms include Simcyp (Certara), GastroPlus (Simulations Plus), and PK-Sim (Open Systems Pharmacology). Among these, PK-Sim is open source and freely available, and FDA reviewers have evaluated PBPK submissions generated using these platforms [35].

Known interactions between posaconazole and tacrolimus are mediated primarily by posaconazole’s potent inhibition of CYP3A enzymes, which markedly reduces tacrolimus metabolism and thereby substantially elevates tacrolimus blood concentrations, increasing the risk of adverse effects. The interaction is well-characterized and clinically significant, and therefore warrants careful management during therapy. Currently, investigations into the interaction between posaconazole and tacrolimus are predominantly retrospective clinical analyses. In vivo pharmacokinetic data are scarce, with only a single reported study, and no studies have evaluated the impact of CYP3A5 genetic polymorphism on the magnitude of the interaction. Although physiologically based pharmacokinetic (PBPK) modeling has advanced and several PBPK models have been developed for TAC or POSA in combination with other drugs, no model to date has specifically simulated TAC pharmacokinetics during co-administration with POSA across different CYP3A5 genotypes. Given the pivotal role of CYP3A5 polymorphism in TAC metabolism and the marked interpopulation variability in CYP3A5 expression, this study provides the first systematic in vitro assessment of posaconazole’s inhibitory effect on CYP3A5, aiming to furnish experimental evidence for individualized mechanisms underlying the interaction.

We developed a PBPK DDI model of TAC and POSA in renal transplant recipients. The model simulated whole-blood TAC trough concentrations across CYP3A5 genotypes and dosing regimens, providing quantitative support for TAC–POSA co-administration. We found that the magnitude of the POSA–TAC interaction depended on the CYP3A5 genotype. In CYP3A5*3*3 nonexpressers, POSA co-administration led to higher TAC C_max_ and AUC than observed in CYP3A5*1 carriers, indicating a need for larger dose reductions to achieve target trough concentrations. PBPK modeling also enables extrapolation from healthy volunteers to special populations (e.g., pediatric, elderly, and pregnant populations, and patients with hepatic or renal impairment), quantification of inter-individual variability (e.g., age, sex, ethnicity, and genetic polymorphisms), and optimization of dosing regimens under specific exposure targets.

When constructing virtual renal transplant recipients, it is important to recognize that many such patients meet the criteria for chronic kidney disease and typically have reduced GFR. Even with good graft function, GFR with a single transplanted kidney often does not fully reach the combined function of two native kidneys [36,37]. In PK-Sim, transplant-specific physiology cannot be directly specified; therefore, we represented post-transplant physiology by selecting the CKD population and setting eGFR to 31.22 mL/min/1.73 m^2^. By setting the target eGFR value, the corresponding individual GFR, kidney volume, renal blood flow, plasma protein binding rate, hematocrit, gastric emptying time and intestinal transit time will change accordingly. We then simulated genotype-specific TAC dose-adjustment strategies during POSA co-administration.

CYP3A5 polymorphism, particularly the 3 allele, is a major determinant of TAC clearance and DDI magnitude [28,38,39]. Prior PBPK studies have demonstrated genotype-dependent interactions between TAC and CYP3A inhibitors (e.g., letermovir and voriconazole) in transplant populations [28,38]. Given the high prevalence of CYP3A5*3 in Chinese individuals and the substantial contribution of CYP3A5 to TAC metabolism [40], characterizing POSA inhibition of CYP3A5 is clinically relevant. To our knowledge, POSA inhibition of CYP3A5 has not been well characterized. Therefore, we quantified POSA inhibition of CYP3A5 using recombinant enzymes in vitro and observed stronger inhibition of CYP3A5 than CYP3A4 in our assays. Gong et al. also conducted in vitro metabolism experiments in the second-generation triazole antifungal drug voriconazole, and found that voriconazole had a stronger inhibitory effect on CYP3A5 than CYP3A4, which was consistent with the results of this study. These data provide parameter support for future PBPK/DDI evaluations of TAC with POSA.

Tacrolimus requires routine therapeutic drug monitoring for dose adjustment to reduce the occurrence of rejection and adverse reactions. It is known that the area under the curve (AUC) is the most accurate index for reflecting the systemic exposure of drugs, but the trough concentration (C_min_) is generally used as the core monitoring parameter of TDM in clinical practice. This is mainly based on the following reasons: First, the determination of AUC requires intensive blood collection, which is expensive and has low patient compliance. Secondly, C_min_ is used as the decision-making basis for dose adjustment in international clinical guidelines and most studies. Finally, a large number of studies have confirmed that tacrolimus C_min_ has a good correlation with AUC_0–12h_, making it a reliable and practical alternative indicator for assessing overall drug exposure. According to the existing tacrolimus TDM guidelines, the monitoring index is the tacrolimus trough concentration. It is recommended that the target trough concentration maintenance level at each time period after surgery is 8–15 ng/mL at 0–3 months, 6–12 ng/mL at 4–6 months, 5–10 ng/mL at 7–12 months, and 5–9 ng/mL at >12 months. The primary monitoring metric is the whole-blood trough concentration. Target C_min_ ranges vary by time post-transplant, and trough sampling is practical and correlates with systemic exposure, supporting dose individualization [41]. We simulated TAC C_min_ under POSA co-administration across genotypes and iteratively adjusted TAC dosing to maintain Cmin within target ranges.

We observed that predicted TAC exposure varied with the duration of POSA co-administration, which is consistent with POSA accumulation before the steady state. This is consistent with POSA’s long half-life and concentration-dependent CYP3A inhibition [42]. Food intake may further affect both drugs: POSA absorption is food dependent, and fasting administration can markedly reduce exposure [43], whereas food can reduce and delay TAC absorption and increase variability [44]. Therefore, consistent administration conditions (e.g., consistently fasting) are important when interpreting TAC TDM and adjusting doses during POSA co-therapy.

This study has several limitations. First, the TAC–POSA DDI model was verified against clinical data only in CYP3A5 nonexpressers, because no corresponding clinical DDI dataset in expressers was available. Second, the model has not been externally validated. Future work will compare model-predicted whole-blood TAC C_min_ with TDM data from our center to further evaluate predictive performance. And the results of this study reflect the contribution of the reversible inhibition component, and the actual DDI risk may be underestimated if a time-dependent mechanism is present. In the future, we will continue to improve the relevant experiments to evaluate the inhibition mechanism.

In summary, we developed a TAC–POSA PBPK DDI model in renal transplant recipients, predicted whole-blood TAC trough concentrations during POSA co-administration across CYP3A5 genotypes, and proposed genotype-informed TAC dose-adjustment strategies. These results provide a quantitative basis for clinical dose modification during TAC–POSA co-therapy.

## Figures and Tables

**Table 1:** Parameters used to establish the PBPK model for tacrolimus.

Parameters	Tacrolimus	Reference
Physiological parameters		
Molecule weight	804.02	Drugbank
PKa value	Base 2.9	Drugbank
	Acid 9.96	
LogP	2.99	Parameter identification
Solubility (PH), mg/mL	0.01 (7.0)	[21]
Fraction unbound	0.01	[22]
Absorption		
Specific intestinal permeability (cm/s)	7.57 × 10^−7^	[23]
Distribution		
Partition coefficients	Berezhkovskiy	
Cellular permeability	PK-Sim standard	
Metabolism		
Reference concentration (μmol/L)		
CYP3A4	3.72 (Caucasian)/3.35 (Asian)	[23]
CYP3A5		
Expressors	0.60 (Caucasian)/0.53 (Asian)	[23]
Nonexpressers	0.04	
Systematic biotransformation		
CYP3A4 K_m_ (μmol/L)	0.21	Parameter identification
CYP3A4 K_cat_ (1/min)	0.43	Parameter identification
CYP3A5 K_m_ (μmol/L)	0.21	Parameter identification
CYP3A5 K_cat_ (1/min)	0.92	Parameter identification
Weibull equation		
Dissolution time (min)	18.85	Parameter identification
Dissolution shape	0.04	Parameter identification

**Table 2:** Parameters used to establish the PBPK model for posaconazole.

Parameters	Posaconazole	Reference
Physiological parameters		
Molecule weight	700.8	Drugbank
PKa value	Base 3.6	Drugbank
	Base 4.6	
LogP	4.58	Parameter identification
Solubility (PH), mg/mL	9.80 × 10^−4^ (7.0)	[25]
Fraction unbound	0.02	[26]
Absorption		
Specific intestinal permeability	5.05 × 10^−5^ cm/min (suspension) 4.80 × 10^−5^ cm/s (tablet)	[17]
Distribution		
Partition coefficients	Poulin and Theil	
Cellular permeability	PK-Sim standard	
Systematic biotransformation		
UGT1A4 K_m_ (μmol/L)	15.90	[17]
UGT1A4 K_cat_ (1/min)	16.52	[17]
Inhibition		
CYP3A4 K_i_ (μmol/L)	5.22 × 10^−3^	[17]
CYP3A5 K_i_ (μmol/L)	3.84 × 10^−3^	In vitro enzymology experiment

**Table 3:** The clinical pharmacokinetic studies used to develop and evaluate the PBPK model of tacrolimus.

Dosing Regimen	Gender (Male%)	Age (Year)	Weight (kg)	Genotype	Reference
TAC model development					
0.015 mg/kg/d	58	44.6 ± 19.1	-	-	[29]
3 mg/d	75	29 (20–44)	78.6 (56–90)	-	[30]
7 mg/d	75	29 (20–44)	78.6 (56–90)	-	[30]
10 mg/d	75	29 (20–44)	78.6 (56–90)	-	[30]
TAC model validation					
5 mg/d	42	30.8 ± 9.9	71.6 ± 19.9	CYP3A5 expressers	[31]
5 mg/d	42	23.5 ± 3.5	66.5 ± 13.5	CYP3A5 nonexpressers	[31]
1 mg/d	100	27.1 ± 7.3	66.7 ± 6.8	CYP3A5 expressers	[32]
1 mg/d	100	27.1 ± 7.3	66.7 ± 6.8	CYP3A5 nonexpressers	[32]

**Table 4:** Clinical studies with information on dosing regimens: simulated and observed AUC_0-∞_ and C_max_ ratios of tacrolimus.

Dosing Regimen	CYP3A5 Genotype	Tacrolimus						Reference
		C_max_ (μmol/L)			AUC0-∞ (μmol/min/L)			
		Predicted	Observed	Ratio	Predicted	Observed	Ratio	
0.015 mg/kg/d	-	0.04	0.03	1.33	19.88	22.19	0.90	[29]
3 mg/d	-	0.02	0.02	1.00	9.15	10.42	0.88	[30]
7 mg/d	-	0.04	0.03	1.33	22.90	23.20	0.99	[30]
10 mg/d	-	0.06	0.05	1.20	35.56	32.56	1.09	[30]
5 mg/d	Expressers	0.02	0.02	1.00	10.41	9.36	1.11	[31]
5 mg/d	Nonexpressers	0.04	0.03	1.33	21.95	15.21	1.44	[31]
1 mg/d	Expressers	0.00407	0.0026	1.56	1.29	0.69	1.87	[32]
1 mg/d	Nonexpressers	0.0071	0.0057	1.24	2.90	1.70	1.70	[32]

**Table 5:** Summary of the predicted vs. observed PK parameters for co-administered drugs.

Drug	PK Parameters	Predicted	Observed	Ratio (Predicted/Observed)
Midazolam+/−Posaconazole	C_max_ Ratio	2.50	2.00	1.25
	AUC_0−t_ Ratio	5.88	4.66	1.26
Voriconazole+/−Tacrolimus	C_max_ Ratio	2.00	2.50	0.8
	AUC_0−t_ Ratio	5.15	4.59	1.12
Tacrolimus+/− Posaconazole	C_max_ Ratio	2.00	2.00	1.00
	AUC_0−t_ Ratio	4.99	4.64	1.08

**Table 6:** Simulation of recommended doses for tacrolimus in patients with different CYP3A5 genotypes.

Dosing Regimen	CYP3A5 Genotype	Tacrolimus	
		C_max_ (ng/mL)	C_min_ (ng/mL)
TAC 0.075 mg/kg/d	Expresser	9.96	2.20
TAC 0.075 mg/kg/d + POSA 200 mg tid	Expresser	23.42	8.15
TAC 0.075 mg/kg/d	Nonexpresser	17.20	4.49
TAC 0.075 mg/kg/d + POSA 200 mg tid	Nonexpresser	32.00	12.26
TAC 0.1 mg/kg/d	Expresser	13.79	3.02
TAC 0.1 mg/kg/d + POSA 200 mg tid	Expresser	32.48	10.7
TAC 0.1 mg/kg/d	Nonexpresser	23.74	6.07
TAC 0.1 mg/kg/d + POSA 200 mg tid	Nonexpresser	43.87	16.04
TAC 0.15 mg/kg/d	Expresser	22.14	4.65
TAC 0.15 mg/kg/d + POSA 200 mg tid	Expresser	52.17	15.65
TAC 0.15 mg/kg/d	Nonexpresser	32.83	9.31
TAC 0.15 mg/kg/d + POSA 200 mg tid	Nonexpresser	70.01	23.31
TAC 0.2 mg/kg/d	Expresser	31.18	6.41
TAC 0.2 mg/kg/d + POSA 200 mg tid	Expresser	74.12	20.46
TAC 0.2 mg/kg/d	Nonexpresser	49.86	12.81
TAC 0.2 mg/kg/d + POSA 200 mg tid	Nonexpresser	98.38	30.31
TAC 0.25 mg/kg/d	Expresser	40.78	8.24
TAC 0.075 mg/kg/48 h + POSA 200 mg tid	Nonexpresser	23.78	4.50
TAC 0.1 mg/kg/48 h + POSA 200 mg tid	Nonexpresser	32.63	5.94
TAC 0.15 mg/kg/48 h + POSA 200 mg tid	Nonexpresser	52.72	8.76
TAC 0.2 mg/kg/48 h + POSA 200 mg tid	Nonexpresser	75.92	11.51

## Data Availability

Data presented in this study is contained within the article and Supplementary Material. Further inquiries can be directed to the corresponding authors.

## Supplementary Materials

The following supporting information can be downloaded at: https://www.mdpi.com/article/10.3390/pharmaceutics18060639/s1, Figure S1: Curves for optimizing incubation time and CYP3A4 enzyme concentration (n = 3); Figure S2: Curves for optimizing incubation time and CYP3A5 enzyme concentration (n = 3); Figure S3: Concentration-dependent metabolism of tacrolimus by recombinant CYP3A enzymes; Figure S4: Simulations of blood concentration-time profiles of tacrolimus after TAC 0.05 mg/kg (A), POSA 400mg bid+TAC 0.05 mg/kg (B) in CYP3A5 nonexpressers; Figure S5: Goodness-of-fit plot for the model of tacrolimus; Table S1: Frequency of the CYP3A5 *1 allele in different race and ethnicity (Birdwell et al., 2015), including assumed activity relative to homozygous carriers of the *1 allele.
