# Supplementary material for: Prediction of Tacrolimus–Posaconazole Interactions in Renal Transplant Patients with Different CYP3A5 Genotypes, Based on Physiological Pharmacokinetic Models
Source: Pharmaceutics. 2026 May 22;18(6):639. doi: 10.3390/pharmaceutics18060639 (PMC13304629; doi:10.3390/pharmaceutics18060639)
Supplement: Supplementary file 1 [file pharmaceutics-18-00639-s001.zip › pharmaceutics-4273268-supplementary.pdf]

pharmaceutics

MDPI

Supplementary Material

Method

1. LC-MS/MS Detection Method

Whole-blood samples were analyzed using a Jasper high-performance liquid chromatography system coupled with a Triple Quad 4500MD triple-quadrupole mass spectrometer (SCIEX, USA), with separation performed on a Kinetex C18 column (3 mm × 100 mm, 2.6 μm; Phenomenex, USA). The MRM transitions used for quantification were m/z 821.2→768.6 (TAC) and m/z 809.5→756.6 (ascomycin). TAC was quantified using ascomycin as the internal standard. A 50 μL whole-blood aliquot was transferred into a 1.5 mL microcentrifuge tube. Internal standard working solution (5 μL) and methanol–acetonitrile (90:10, v/v; 100 μL) were added; this was followed by vortex-mixing for 3 min and centrifugation at 16,400 rpm for 10 min. A 50 μL aliquot of the supernatant was transferred to a second tube, diluted with 50 μL water, centrifuged again, and then transferred to an autosampler vial for LC-MS/MS analysis.

The retention times for tacrolimus and the internal standard ascomycin were 3.12 min and 3.13 min, respectively. Endogenous substances in whole blood did not interfere with the determination of the analytes. The method demonstrated good linearity for TAC over the concentration range of 0.5–100 ng/mL (r2>0.99). The accuracy ranged from 90.53% to 108.78%, with precision (RSD)≤15%. The extraction recovery was 71.48%–94.88% (RSD: 5.72%–13.75%), and the matrix effect was 97.04%–104.82% (RSD: 5.07%–8.99%). All stability results met the required methodological criteria.

2. Determination of Optimal Incubation Time and Enzyme Amount for Tacrolimus with Recombinant Human CYP3A4/5

Tacrolimus was tested at final concentrations of 1.0 μM and 0.5 μM. The substrate was incubated with a series of CYP3A4/5 enzyme concentrations (final concentrations: 2, 5, and 10 pmol/L) on an oscillating shaker at 37°C for 0, 5, 10, 15, 20, and 30 minutes. The remaining amount of substrate was plotted against the incubation time. The optimal incubation time was selected as the period demonstrating a linear relationship with time, where substrate metabolism did not exceed 20%. Similarly, the optimal microsomal enzyme concentration was selected as the concentration showing a linear relationship with enzyme amount while keeping substrate metabolism below 20%.

3. Determination of the Km Value for Tacrolimus Incubated with Recombinant Human CYP3A4/5

A master mix was prepared by combining PBS buffer, the G-6-P solution, the G-6-PD solution, and the $MgCl_2$ solution in a ratio of 9:2:2:2. Aliquots of 150 μL of this mixed solution were dispensed into individual EP tubes. Then, 20 μL of the CYP3A4/5 enzyme was added, followed by 10 μL of the tacrolimus test solution (yielding final concentrations of 0, 0.15, 0.3, 0.75, 1.5, 3, and 7.5 μM). The mixture was vortexed and pre-incubated in a 37°C water bath for 5 minutes. A separately prepared β-NADP solution was also prewarmed for 5 minutes. The reaction was initiated by adding 20 μL of a 10 mmol/L β-NADP solution, and the tubes were immediately returned to the 37°C water bath. After incubating for 10 min and 5 min (for the respective substrate concentrations), the reactions were terminated by adding 400 μL of ice-cold methanol. Subsequently, 5 μL of the internal standard solution was added. The termination mixture was vortexed for 1 minute to ensure thorough mixing; this was followed by centrifugation prior to injection for analysis.

Results

1. Tacrolimus incubation time and the amount of recombinant human CYP3A4/5 enzyme substance

After optimizing the incubation times and the amounts of enzyme substance, the amount of CYP3A4 enzyme substance was 2 pmol and the associated incubation time was 10 min, and the amount of CYP3A5 enzyme substance was 2 pmol and the associated incubation time was 5 min.

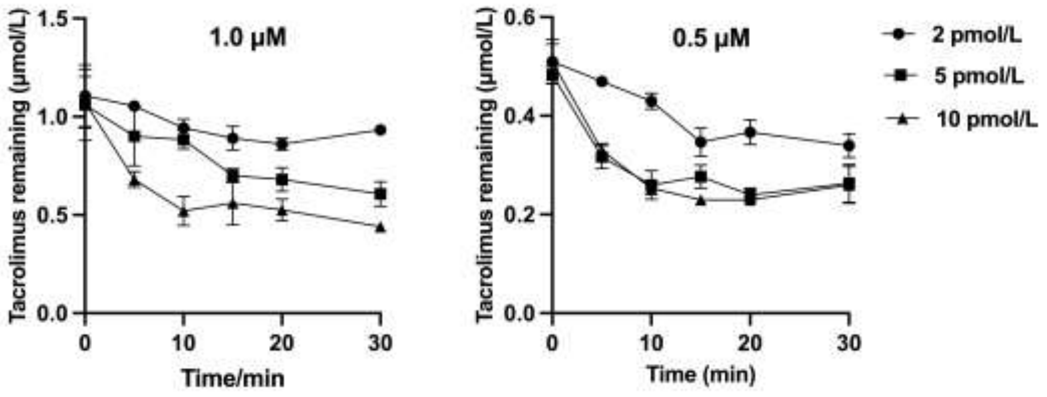

Figure S1. Curves for optimizing incubation time and CYP3A4 enzyme concentration (n = 3).

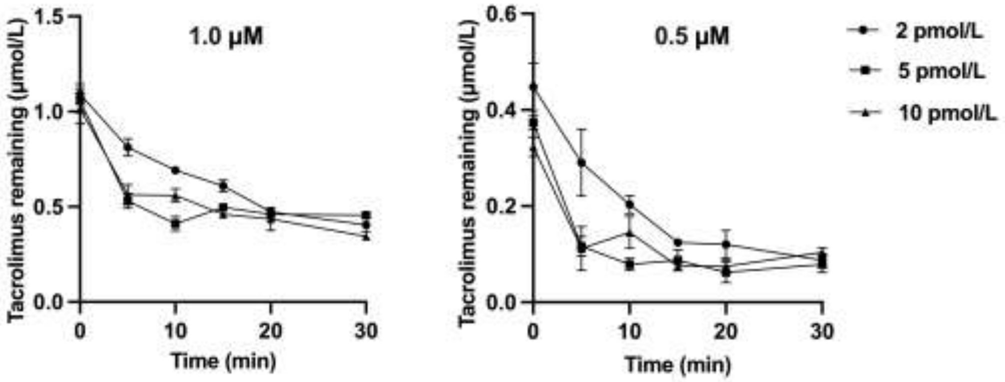

Figure S2. Curves for optimizing incubation time and CYP3A5 enzyme concentration (n = 3).

2. Determination of the Km Value of Tacrolimus for Recombinant Human CYP3A4/5 Enzymes

Tacrolimus is a substrate for both CYP3A4 and CYP3A5 enzymes and is metabolized by recombinant human CYP3A enzyme systems. The metabolism of tacrolimus follows nonlinear kinetics, which conform to the Michaelis–Menten equation. The metabolic velocity versus substrate concentration curve was plotted using GraphPad Prism 10.2.1 software. Subsequently, the Michaelis–Menten equation was applied to calculate the Km value for tacrolimus metabolism. The calculated Km values were 1.438 μM for CYP3A4 and 1.199 μM for CYP3A5.

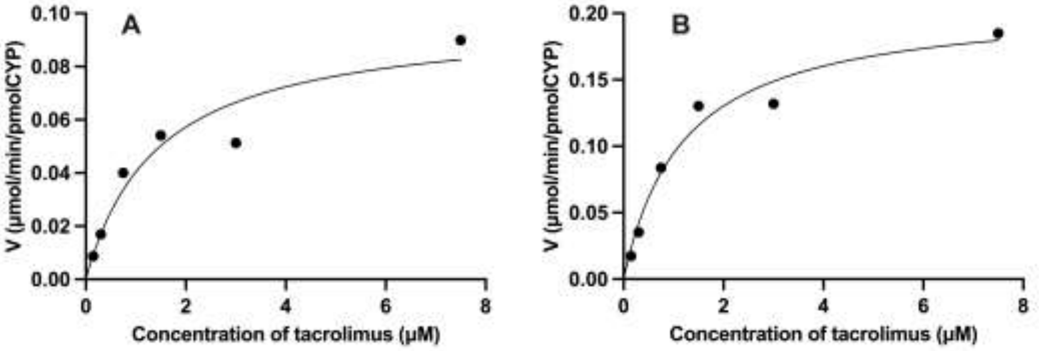

Figure S3. Concentration-dependent metabolism of tacrolimus by recombinant CYP3A enzymes.

The metabolic velocity is plotted against the substrate concentration. Data are from three independent experiments. (A) metabolism by CYP3A4. (B) metabolism by CYP3A5.

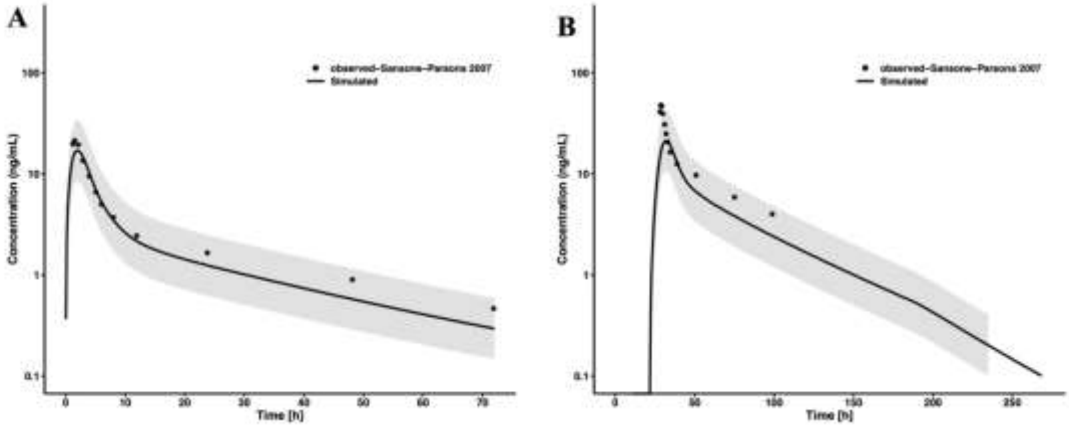

Figure S4. Simulations of blood concentration–time profiles of tacrolimus after TAC 0.05 mg/kg (A), POSA 400mg bid+TAC 0.05 mg/kg (B) in CYP3A5 nonexpressers.

3. Tacrolimus PBPK model development

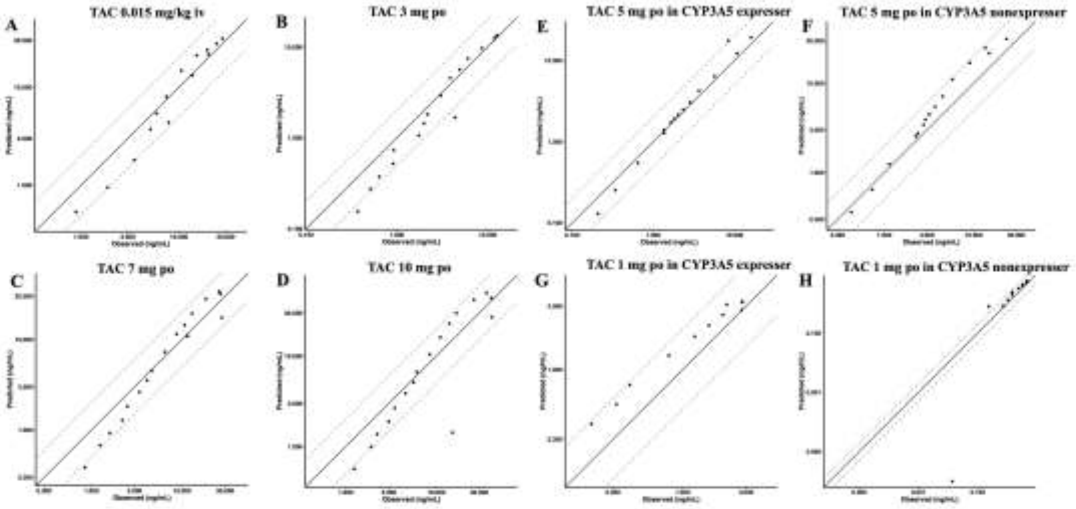

Figure S5. Goodness-of-fit plot for the model of tacrolimus.

Observed concentrations (Observed) versus population model-predicted concentrations (Predicted). The black solid line represents the line of identity (y=x), and the grey dashed lines indicate the 2-fold deviation range. A: Tacrolimus 0.015 mg/kg intravenously; B, C, D: tacrolimus 3, 7, 10 mg orally. E, G: Oral tacrolimus in the CYP3A5 expressers; F, H: oral tacrolimus in the CYP3A5 nonexpressers.

Table S1. Frequency of the *CYP3A5 *1* allele in different races and ethnicities (Birdwell et al., 2015), including assumed activity relative to homozygous carriers of the *1 allele.

Race and Ethnicity	Frequency of the CYP3A5 *1 allele, %	Assumed activity, %
African American	60.5	60.5
Asian	25.8	25.8
Latin American	20.2	20.2
Caucasian	7.8	7.8
